# To Synthesize Hydroxyapatite by Modified Low Temperature Method Loaded with *Bletilla striata* Polysaccharide as Antioxidant for the Prevention of Sarcopenia by Intramuscular Administration

**DOI:** 10.3390/antiox10030488

**Published:** 2021-03-20

**Authors:** Ya-Jyun Liang, Jia-Yu Hong, I-Hsuan Yang, Xin-Ran Zhou, Yi-Wen Lin, Tzu-Chieh Lin, Chun-Han Hou, Feng-Huei Lin

**Affiliations:** 1 Institute of Biomedical Engineering, College of Medicine and College of Engineering, National Taiwan University, Taipei 10617, Taiwan; d04548016@ntu.edu.tw (Y.-J.L.); r04548053@ntu.edu.tw (J.-Y.H.); f07528014@ntu.edu.tw (I.-H.Y.); r07528061@ntu.edu.tw (X.-R.Z.); d06548012@ntu.edu.tw (Y.-W.L.); d05548018@ntu.edu.tw (T.-C.L.); 2 Department of Orthopedic Surgery, National Taiwan University Hospital, Taipei 10617, Taiwan; chhou@ntu.edu.tw; 3 Division of Biomedical Engineering and Nanomedicine Research, National Health Research Institutes, No. 35, Keyan Road, Miaoli County 35053, Taiwan

**Keywords:** sarcopenia, antioxidant, *Bletilla striata* polysaccharide (BSP), hydroxyapatite, drug delivery

## Abstract

Oxidative stress has been suggested as an important factor in the progress of sarcopenia. The current treatments for sarcopenia have the disadvantages of insufficient effect or daily administration. Therefore, an alternative for effective, safety and long-term treatment may be a solution for unmet needs. *Bletilla striata* polysaccharide has been reported to have anti­oxidative and anti-inflammatory properties. In this study, we used *Bletilla striata* polysaccharide (BSP) combined with hydroxyapatite, a carrier. We hypothesized that the resulting combination (BSP-HAP) is a good formula for the controlled release of BSP via intramuscular (IM) administration, so as to prevent the worsening of presarcopenia or even recover from the early stage of the illness. In this research, BSP-HAP was synthesized by a modified low temperature co-precipitation process that would be beneficial for BSP loading. By conducting DCFDA, WST-1 and the Live/Dead assay, BSP-HAP is shown to be a biocompatible material which may release BSP by cells through the endocytosis pathway. Animal studies revealed that the rats treated with BSP-HAP could effectively recover muscle endurance, grip strength or fat/lean mass ratio from lipopolysaccharide (LPS)-induced sarcopenia. This study shows BSP delivered by BSP-HAP system has potential for application in the treatment and prevention of sarcopenia in the future.

## 1. Introduction

Sarcopenia is a syndrome characterized by the progressive loss of skeletal muscle mass, strength, and endurance with a risk of adverse outcomes such as physical disability, poor quality of life and even death [1]. The loss of muscle mass and strength is a serious risk and may lead to a disability in the aging population; such disability would worsen the health problems associated with sarcopenia [2]. In the elderly, the rate of muscle breakdown and synthesis may not be able to sustain healthy muscle mass due to a progressive decrease in skeletal muscle mass. According to previous studies, muscle dysfunction, which has been linked to sarcopenia, along with the loss of muscle mass, involves not only contractile impairment but also metabolic and endocrine abnormalities. These abnormalities, including chronic elevated low-grade systemic inflammation, systemic inflammation, inflame-aging and oxidative stress, would negatively affect the metabolism and the immune system of the host [3,4]. Aging has been associated with oxidative stress and chronic inflammation in muscle [5]. A previous study has suggested that oxidative stress and low-grade inflammatory state affects muscle protein metabolism, mitochondrial dysfunction, apoptosis, and induce sarcopenia [6].

Several methods, including exercise, dietary supplementation, and hormonal interventions, have been proposed to delay the progression of sarcopenia [7,8]. However, none of the methods markedly delayed the progression of sarcopenia. Despite exercise being a common method for counteracting sarcopenia, the complex nature of the syndrome combined with many variables such as age and disease makes patients unable to exercise regularly [8]. Although elders require more proteins achieving nitrogen balance, clinicians were reported to be hesitant in recommending high-protein diets for the elderly due to the potentially harmful effects of the diet on renal and cardiovascular function [9]. The effects of hormonal interventions such as testosterone supplementation on sarcopenia have also been reported as testosterone and other adrenal androgens decline with age, and epidemiological studies support a relationship between hormonal deficiencies and muscle strength. However, treatment with high doses of testosterone has been reported to cause cancer, particularly that of the prostate [10]. These interventions have shown promising results in treating or preventing sarcopenia; however, they also have shown side effects. Therefore, an alternative for effective, safety and long-term treatment or prevention of sarcopenia may be necessary.

The European Working Group on Sarcopenia in Older People (EWGSOP) has proposed the progressive stages of sarcopenia: presarcopenia, sarcopenia, and severe sarcopenia. Presarcopenia is characterized by low muscle mass without reduction in muscle strength or physical performance [11]. A person with presarcopenia may fully recover if given adequate treatment. In the present study, we used a polysaccharide extracted from a traditional herb and combined it with a biodegradable ceramic by intramuscular injection (IM) as a formula for the controlled release of the polysaccharide that would prevent presarcopenia from worsening or improve recovery from presarcopenia.

*Bletilla striata (Thunb.) Reichb. f. (Orchidaceae)* is a traditional herbal medicine. In recent studies, a polysaccharide extracted from *Bletilla striata* (BSP) has been reported to have anti­oxidative and anti-inflammatory properties [12,13,14]. BSP is an effective scavenger of reactive oxygen species (ROS) and can downregulate the expression of inflammatory factors such as tumor necrosis factor α (TNF-α) and interleukin 6 (IL-6) [15,16]. However, the BSP is a water-soluble gel and is not biostable in physiological environments. It must be combined with a carrier to achieve a controlled and long-term release for at least 2 weeks. Hydroxyapatite (HAP) is a major mineral in the bones and teeth of the human body and has good biocompatibility and biodegradability. HAP has been widely used in drug delivery systems although its release was not easily controlled in initial studies; many researchers were troubled for decades due to its limited applications [17,18]. In the present study, porous HAP with wide surface area and space was synthesized for BSP loading; the combined substance is abbreviated as BSP-HAP hereon. We aimed to test the hypothesis that BSP-HAP delivered by IM injection stays in muscle tissues, is gradually assimilated by defense cells such as macrophages via endocytosis, and that the endosome with BSP-HAP later coalesces with a lysosome as an endosome/lysosome complex, wherein the HAP is fully dissolved. Once HAP is dissolved, the endosome/lysosome complex would be broken down by the osmotic pressure from the increase in Ca^2+^ and PO_3_^4−^ concentration in the complex. Through this mechanism, BSP would be released into the cytoplasm and then into the extracellular space due to high Ca^2+^ levels. Afterwards, BSP would then enter vascular circulation by diffusion and be transported throughout the body [18,19,20]. In addition, the biodegraded products of BSP-HAP would be Ca^2+^, PO_3_^4−^, and glucose. Among them, the calcium ions may improve muscle functions, and glucose will be a source of energy [21,22].

**Figure 1:**
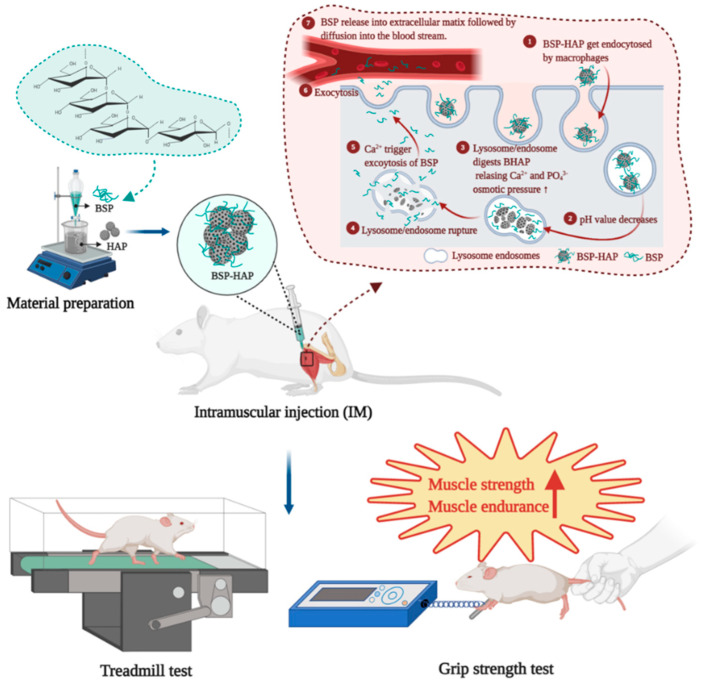
The developed *Bletilla striata* and hydroxyapatite (BSP-HAP) particle preparation and in vivo delivery via intramuscular (IM) injection. Shown are the sequences of BSP-HAP particle synthesis. In addition, the method of IM injection of BSP-HAP particles is indicated and so are the multiple steps and mechanisms involved for the BSP delivery to the blood circulation system.

In the present study, the extracted and synthesized materials were characterized by nuclear magnetic resonance (NMR) imaging, Fourier-transform infrared (FTIR) spectroscopy, X-ray diffraction (XRD) imaging, scanning electron microscopy (SEM), and energy dispersive spectrophotometry (EDS) to identify the molecular structure, functional groups, crystal structure, morphology, and chemical composition, respectively, of BSP in BSP-HAP. The particle size of the BSP was measured by using a Zetasizer. Thermogravimetric analysis (TGA) was used to determine the loading efficiency of BSP. The cell viability and cytotoxicity of BSP were evaluated by using WST-1 assay and LIVE/DEAD staining, respectively. The BSP release profile in vitro was evaluated in neutral and acidic conditions to mimic the physiological and lysosomal environment, respectively. The internalization of BSP-HAP by defense cells was examined by using confocal microscopy and fluorescence staining. SD rats with lipopolysaccharide (LPS)-induced sarcopenia were used as the animal model for the study. Treadmill test, grip strength test, magnetic resonance imaging (MRI), and histology sectioning were used to evaluate the efficacy of BSP-HAP on sarcopenia. The safety of BSP-HAP was evaluated by using blood element analysis and serological analysis. The constant release of BSP from BSP-HAP would be achieved by cellular activity of endocytosis process. The overall experimental design was schemed as Figure 1.

## 2. Materials and Methods

### 2.1. Bletilla Striata Polysaccharide (BSP) Extraction and Purification

Dried *Bletilla striata* were obtained from Seng Chang Pharmaceutical Company, Taoyuan, Taiwan. BSP extraction and purification were performed according to a previous study with the following modifications: 100 g of *Bletilla striata* powder was dissolved in 100 mL of ddH_2_O at 80 °C for 4 h [23]. The supernatant was collected; afterwards, 300 mL of 95% ethanol was added into the supernatant. Then, the solution was incubated at 25 °C overnight. The collected precipitate was resuspended in 1800 mL ddH_2_O, and 600 mL Sevage reagent (*n*-butanol/chloroform 1:4) was added into the suspension for deproteinization; the suspension was stirred overnight. Then, the supernatant was collected and dialyzed against ddH_2_O to remove *n*-butanol and lyophilized by vacuum drying to complete the extraction/purification of *Bletilla striata* polysaccharide (BSP).

### 2.2. Preparation of the BSP-HAP

In this study, 0.5 M calcium hydroxide (Ca(OH)_2_, 1305-62-0, Sigma-Aldrich, St. louis, MO, USA) solution was prepared by dissolving 3.86 g Ca(OH)_2_ in 50 mL ddH_2_O and incubating the resulting solution at 50 °C in a water bath. A 1.5% BSP solution was prepared by dissolving in 50 mL water. The BSP solution was added at a rate of approximately 0.1 mL/min into the 0.5 M Ca(OH)_2_ solution and stirring for 2 h. A stoichiometric amount (Ca/P molar ratio = 1.67) of 100 mL 0.3 M phosphoric acid (H_3_PO_4_, 7664-38-2, Sigma-Aldrich) was added drop-wise at a rate of approximately 1 mL/min into the mixture. After the addition, the pH was adjusted to 8.9 to ensure HAP precipitation and the desired morphology. The mixture was stirred for 2 h and aged at 50 °C for 48 h. Finally, the precipitate was collected, washed three times with deionized water, and lyophilized by vacuum drying.

### 2.3. Material Characterization

The molecular structure of BSP was analyzed according to 13C NMR and 1H NMR spectra measurements at 30–50 mg/mL concentrations [21]. Then, the spectra were recorded on a Bruker ARX-600 instrument (600 MHz, Bruker Co., Ltd. Switzerland).

The functional groups of BSP, HAP, and BSP-HAP were analyzed by Fourier transform infrared (FTIR) spectroscopy (JASCO 410, Tokyo, Japan) at 400–4000 cm^−1^ scanning range and 400 nm/min scanning rate.

The crystal structure of the synthesized HAP and BSP-HAP was identified by using an X-ray diffractometer (Rigaku Geigerflex, Tokyo, Japan). XRD patterns were obtained at 30 kV and 15 mA, with the diffraction angle set within the range of 10–70° at a scanning rate of 1°/min [24].

The morphology of the synthesized particles was observed under Scanning electron microscopy (SEM, Philips XL30, Amsterdam, Netherlands) with an accelerating voltage of 15 kV. Particles were mounted on the sample stage of SEM and coated with a platinum film by sputtering physical vapor deposition (PVD). The platinum film was used to increase the imaging resolution and prevent undesired charge accumulation.

The particle sizes of HAP and BSP-HAP were detected by dynamic light scattering (DLS, Malvern, Worcestershire, UK) set at 25 °C. The zeta potential was determined by DLS associated with electrophoretic mobility at pH 7.4. The sample was dispersed in ddH_2_O for measurements of mean size.

The chemical composition of HAP and BSP-HAP was analyzed by using an energy dispersive spectrophotometer (EDS, Bruker, FQ5060) that was integrated into an SEM. The EDS was also used to confirm the ratio between calcium and phosphorus in HAP.

The specific surface area of HAP and BSP-HAP were measured by Brunauer, Emmett, and Teller (BET, Micromeritics ASAP2010, Norcross, GA, USA) using nitrogen gas adsorption−desorption isotherms.

### 2.4. BSP Loading Efficiency and Release Profile

Thermogravimetric analysis (TGA, CAMCOR, Dynamic Q500) was used to determine the amount of BSP in BSP-HAP. A 5 mg sample was placed in an alumina crucible and heated at a rate of 10 °C/min from 20 to 600 °C under nitrogen flux. The reference material used was submicron Al_2_O_3_ powder. The weight lost at certain temperatures was recorded by a computer system. The weight loss at the volatilized temperature of BSP was used to determine how much BSP was in BSP-HAP [13].

In vitro release of BSP was carried out in phosphate buffer solution (PBS) at pH 3 and 7 to mimic the physiological environment and the environment of the lysosome/endosome complex, respectively. In brief, 1 g BSP-HAP was immersed in 50 mL phosphate buffered solutions (PBS) the temperature was maintained at 37 °C for certain periods (Supernatants were quantified the release in the first 2 h, 6 h, 12 h, 24 h, and collect every 2 days over a period of 11 days). Then, the supernatants were collected for further analysis by UV–Vis spectrophotometry (JASCO V-670, Tokyo, Japan) at 226 nm wavelength.

### 2.5. The Evaluation of Cell Viability and Cytotoxicity

The biocompatibility of the composite was tested according to the International Standard ISO 10993. The cell viability of the BSP-HAP was evaluated using the water-soluble tetrazolium (WST-1, Takara) assay in the C2C12 cell line from Bioresource Collection and Research Center, Taiwan. The C2C12 myoblasts were cultured in DMEM medium (Dulbecco’s modified Eagle’s medium, Sigma), 1% Antibiotic-Antimycotic (Gibco), and 10% Fetal bovine serum (FBS, Hyclone) and incubated at 37 °C and 5% CO_2_ in a humidified incubator (Nozaki, Nikai, Okabe, Nagahama and Eto, 2016). Briefly, C2C12 myoblasts were seeded in 96-well plate at a density of 10^4^ cells/well and cultured for 1 day to full adhesion. An extract medium was prepared by 0.2 g/mL the materials were incubation for 24 h at 37 °C. The extracted supernatant was collected for subsequent experiments. Zinc diethyldithiocarbamate (ZDEC, Sigma-Aldrich) and aluminum oxide (Al_2_O_3_, Sigma-Aldrich) were used as positive control and negative control, respectively. The extracted medium from the HAP, BSP-HAP, positive control, and negative control were cultured with C2C12 myoblasts for 24 h. Afterwards, 100 μL WST-1 solution was added into the wells, and the plate was incubated for 2 h. Then, the absorbance values of each well were measured at 450 nm by using an ELISA plate reader (Molecular Devices) [25,26].

Cytotoxicity was evaluated by using LIVE/DEAD staining kit (L3224, Invitrogen) according to the manufacturer’s instructions. An extract medium was prepared with a volume of 0.2 g/mL and the materials were incubation for 24 h at 37 °C. The extracted supernatant was collected for subsequent experiments. The control group included C2C12 cells without treatment. Zinc diethyldithiocarbamate and aluminum oxide were used as positive control and negative control, respectively. The C2C12 myoblasts were seeded in a 24-well plate at a density of 3 × 10^4^ cells/well and incubated for 24 h. Afterwards, extract medium was added into each well of the culture plate. Then, the plate was incubated for 1 day. Living and dead cells were stained with calcein AM and ethidium homodimer-1, respectively. The LIVE/DEAD staining was observed by using a fluorescence microscope (IX81, Olympus).

### 2.6. Detection of Cellular ROS Generation

The ability of BSP-HAP in the suppression of LPS-induced ROS generation was measured by a DCFDA-cellular ROS detection assay kit (ab113851, Abcam). In brief, C2C12 myoblasts were seeded in 96-well plates at a density of 10^4^ cells/well and cultured for 1 day to full adhesion. The medium was then added with 1 mg/mL of HAP and BSP-HAP for 24 h. Next, LPS (1 μg/mL) was added to induce ROS generation for 24 h. The medium was then removed, washed with PBS, and added culture medium with 25 μM DCFDA regent for another 45 min at 37 °C. The fluorescence intensity was detected by multimode microplate readers (Molecular Devices, SpectraMax i3x, USA) with excitation and emission wavelengths of 485 nm and 535 nm, respectively.

### 2.7. Animal Model with Lipopolysaccharide-Induced Sarcopenia Model

Sprague Dawley male rats (12 months of age, 500–600 g) were purchased from Bio LASCO Taiwan Co., Ltd. All animal experiments were performed in accordance with the guidelines of National Taiwan University, College of Medicine, Institutional Animal Care and Use Committee (IACUC, No. 20180117).

The experiments were conducted over a period of 2 months. The LPS (L2880, Sigma) was dissolved in normal saline before injection. Sarcopenia with muscle injury was induced by using LPS IP injection twice a week based on previous studies [27,28]. The SD rats were randomly divided into three groups of six: (1) the control group was intraperitoneally (IP) injected with 200 μL sterile saline; (2) an LPS group was IP injected with LPS (150 μg/kg BW) twice a week to induce sarcopenia; (3) an LPS-BSP-HAP group intramuscularly (IM) injected with 150 mg/kg BSP-HAP at 0, 14, 28 and 42 days as treatment before IP injected with LPS. The rats were subjected to treadmill and grip strength tests before euthanasia. After euthanasia, rat muscle was harvested for MRI examination, and whole blood was collected to evaluate the safety.

### 2.8. Treadmill Test and Grip Strength Test

The muscle endurance of SD rats was evaluated by using treadmill test (Exer-6M Treadmill; Columbus Instruments, Ohio, USA). All rats were firstly trained to run for 1 week, by running 30 min on a treadmill at a starting speed of 2 m min^−1^ and ending at 20 m min^−1^ (2 m min^−1^ increase). The bottom of the treadmill was defined as low performance sections and equipped with an electrical stimulation component. Muscle endurance was determined by measuring the time of the rats stayed in low performance sections of the treadmill during the 30-min test [29].

The muscle function of rats was evaluated by using grip strength test. Grip strength was measured by using a grip strength meter (Columbus Instruments, Columbus, OH, USA) according to a previous report [30]. The rat’s paws were placed on a wire grid, and the rat’s tail was pulled backward; the maximum strength of the grip was recorded according to the strongest grip of the rat onto the wire grid. Each rat was tested 6 times with an interval of 5 min. The results are expressed in grams.

### 2.9. Fat-to-Muscle Ratio Determination by Magnetic Resonance Imaging (MRI)

MRI was used to examine muscle morphology and to measure fat-to-lean mass ratio. After the euthanasia of rats, their hind legs were harvested and subsequently mounted onto Bruker Biospec 7T MRI (Bruker Corporation, Billerica, MA, USA). The framed parameters were set as TE = 3.776 ms, TR = 25 ms, FA = 30°, NA = 1, MTX = 256 × 256 × 128, and resolution = 110 × 110 × 400 μm. Fat-to-lean mass ratio were analyzed by using ImageJ software.

### 2.10. Blood Biochemical Analysis

The safety of BSP-HAP in vivo was evaluated by blood element analysis and serological analysis. Blood was collected after euthanasia by cardiac puncture. Serum was obtained and centrifuged at 1300× *g* at 4 °C for 15 min. The collected serum was stored at −80 °C. For the biochemical tests, total protein (TP), creatine kinase (CK), lactate dehydrogenase (LDH) and calcium (Ca) in serum were measured. Reference: Charles River Laboratories, CD^®^ IGS Rat Model Information Sheet [31,32].

### 2.11. Statistical Analysis

Statistical data are expressed as mean ± standard deviation (SD). Statistical analysis was performed by using one-way ANOVA; *p*-values (*) less than 0.05 were considered statistically significant.

## 3. Results

### 3.1. Molecular Structure, Functional Groups and Crystal Structure Identification

**Figure 2:**
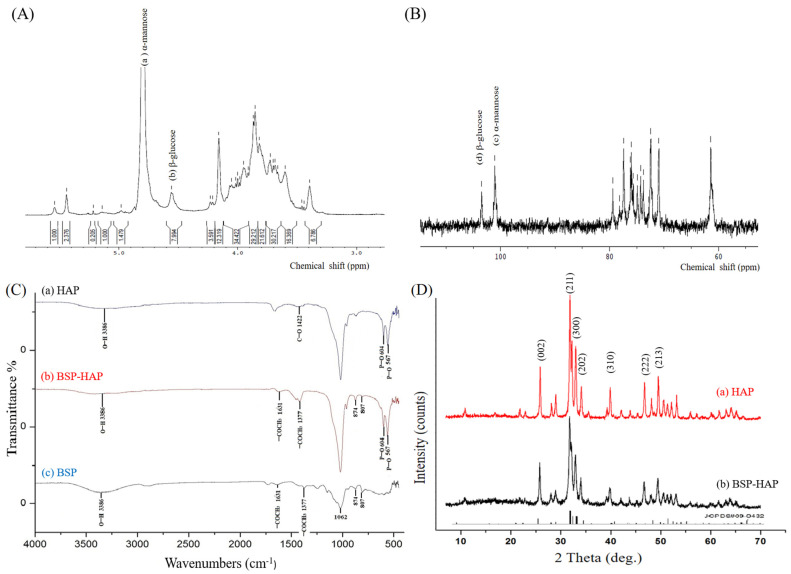
The Molecular structure, functional groups and crystal structure identification of BSP-HAP. (**A**) The ^1^H-NMR spectrum, the peaks at 4.8 ppm and 4.5 ppm indicated as (a) and (b) in the structure are the characteristic peaks for α-mannopyranose and β-glucopyranose, respectively. (**B**) The ^13^C-NMR spectrum, the signals at 101.1 ppm and 103.5 ppm could be attributed to α-mannopyranose and β-glucopyranose as indicated in (c) and (d) in the structure, respectively. (**C**) FTIR patterns of HAP, BSP-HAP, and BSP. The absorption bands at 567 cm^−1^, 604 cm^−1^, and 3386 cm^−1^ were matched to the HAP. Absorption band at 807 cm^−1^, 874 cm^−1^ and 1062 cm^−1^ were matched to the BSP. The FTIR spectrum presents all the characteristic absorption bands contributed by HAP and BSP. (**D**) X-ray diffraction patterns of HAP and BSP-HAP. All the characteristic peaks were matched to the standard pattern of HAP in No. 09-0432 of JCPD card.

The molecular structure of BSP was analyzed by using ^1^H NMR and ^13^C NMR. Shown in Figure 2A is the ^1^H NMR pattern of the extracted BSP, wherein the chemical shifts at 4.8 ppm and 4.5 ppm indicated as (a) and (b) in the structure correspond to the characteristic peaks for α-mannopyranose and β-glucopyranose, respectively. The signals in other chemical shifts in the 3.3–4.2 ppm range belong to non-anomeric ring protons. Shown in Figure 2B is the ^13^C NMR pattern of the extracted BSP. The signals at 101.1 ppm and 103.5 ppm indicated as (c) and (d) in the structure correspond to the characteristic peaks for α-mannopyranose and β-glucopyranose, respectively. The chemical shifts in the 70–80 ppm range were identified to correspond to non-anomeric carbon rings. Our results of ^1^H NMR and ^13^C NMR of BSP were similar to those of previous reports [33]. Altogether, we infer that BSP was extracted and purified from *Bletilla striata*.

Shown in Figure 2C(a) is the FTIR pattern of the synthesized HAP. The absorption bands at 567 cm^−1^, 604 cm^−1^, and 3386 cm^−1^ regions were identified as O−H stretching vibration mode, PO_4_^3−^ bending vibration mode, and PO_4_^3−^ stretching vibrations mode, respectively. The FTIR spectra of BSP are shown in Figure 2C(c). The absorption band at 807 cm^−1^ and 874 cm^−1^ regions correspond to mannose and β-type glycosidic linkage, respectively. The absorption bands at the 3386 cm^−1^ region correspond to hydroxyl groups (O−H), whereas the absorption bands at the 1062 cm^−1^ region correspond to pyranoses. The absorption bands at 1361 cm^−1^ and 1377 cm^−1^ regions correspond to −COCH_3_. Shown in Figure 2C(b) is the FTIR pattern of the synthesized BSP-HAP. The FTIR spectrum presents all the characteristic absorption bands contributed by HAP and BSP.

Figure 2D(a) was the XRD pattern of the synthesized HAP, wherein all the characteristic peaks such as (002), (211), (300), (202), (310), (222), and (213) fully match the standard XRD pattern of HAP with JCPDS card no. 09-0432. Shown in Figure 2D(b) is the XRD pattern of the prepared BSP-HAP. All the characteristic peaks match the XRD pattern of HAP as previously determined. Based on the results, we infer that the crystal structure of synthesized BSP-HAP was exactly the same as that of HAP prepared by the conventional method.

### 3.2. Morphology, Particle Size and Chemical Composition of BSP-HAP

**Figure 3:**
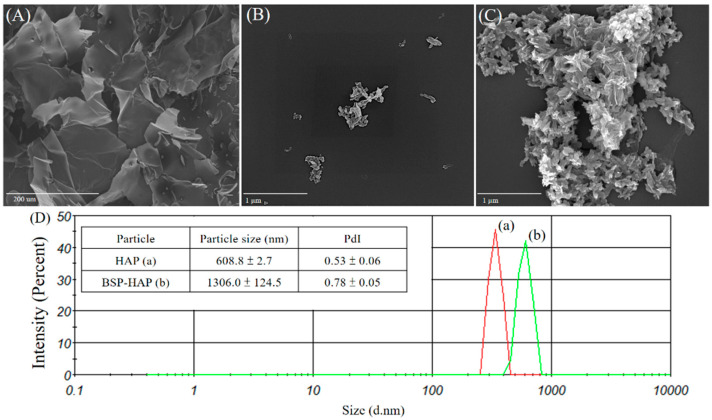
SEM images of (**A**) BSP, (**B**) HAP, and (**C**) BSP-HAP. The morphology of the extracted BSP showed a typical dried hydrogel morphology with many pores left from water-occupied space. The HAP showed a needle-like or flake morphology, and some grains would aggregate into a spherulite structure. The morphology of BSP-HAP was as the morphology of HAP spherulite aggregates and smoothly canopies gel-film on the crystal. The analysis of average particle size and distribution (**D**). The peak (a) was the particle size distribution of HAP; the peak (b) was the particle size distribution of BSP-HAP. The average particle size of HAP and BSP-HAP were in the range of 900−2000 nm. The summary is shown in the table on the left side.

The morphology of BSP, HAP, and BSP-HAP was examined under SEM as shown in Figure 3. Shown in Figure 3A is the morphology of the extracted BSP dried by lyophilization. Moreover, a typical morphology of a dried hydrogel with many pores that were previously occupied by water can be seen. Shown in Figure 3B is the morphology of HAP. A single grain of HAP resembles a needle-like or flake-like morphology and is 300–500 μm in length and 60–100 nm in width. There were lots of pores between the grains that left more surface area and space. In Figure 3C, the morphology of the developed BSP-HAP that was dried by lyophilization is shown. Its morphology resembles a crystal surface covered in gel, which maintains the aggregation into a spherulite structure.

The average particle size and distribution of HAP and BSP-HAP analyzed by using a DLS is shown in Figure 3D and summarized in the attached table. The average particle sizes of HAP and BSP-HAP were determined to be 608.8 nm and 1306.0 nm, respectively, whereas their polydispersity indices (PdI) were 0.53 and 0.78, respectively. The results indicate that the BSP-HAP size was in the 500–1500 nm range, which is suitable for macrophage endocytosis.

The chemical composition of the synthesized HAP and BSP-HAP was analyzed by EDS integrated into an SEM and is shown in Table 1. The non-stoichiometric molar ratio of calcium to phosphorus in HAP ranged from 1.50 to 1.67. The molar ratio of calcium to phosphorus in the synthesized HAP was 1.66, whereas the ratio of BSP-HAP that was developed was 1.55.

The BET analysis was shown in Table 2. The surface area of BSP-HAP was in 60.7645 nm; that was higher than that of HAP prepared by the traditional method, for better BSP loading efficiency.

### 3.3. Determination of the Loading Efficiency of BSP

**Figure 4:**
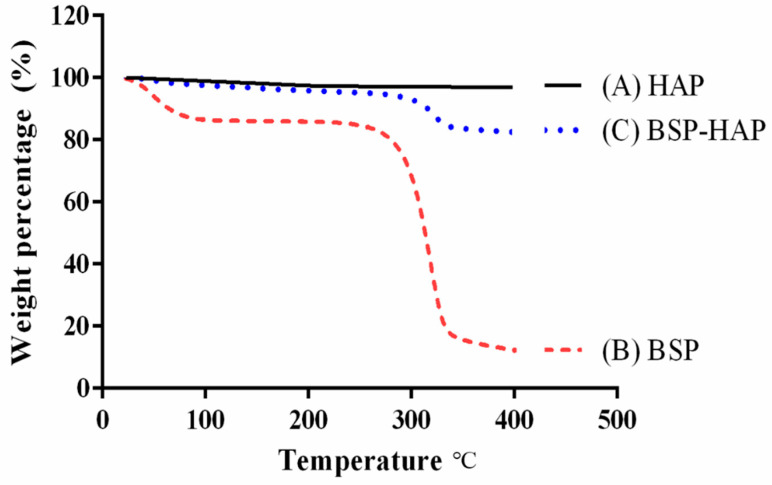
BSP loading efficiency analysis by TGA. The TGA curve of (A) the synthesized HAP, (B) the extracted BSP; where a sharp weight loss at the temperature of 307 °C was indicated as the volatilization temperature of BSP, and (C) the developed BSP-HAP; a weight loss of approximately 13.6% was found at the volatilization temperature of BSP.

Thermogravimetric analysis (TGA) was used to evaluate the loading efficiency of BSP and is shown in Figure 4. The black curve in Figure 4 represents the TGA curve of the synthesized HAP; it shows little weight loss as the temperature was increased from room temperature to 400 °C. The red curve represents the TGA curve of the extracted BSP; it shows a sharp weight loss at 307 °C, which was identified as the volatilization temperature of BSP. The blue curve at the middle of Figure 4 represents the TGA curve of the developed BSP-HAP; a weight loss of approximately 13.6% at 307 °C was due to the loss in BSP.

### 3.4. Release Profile of BSP from BSP-HAP

**Figure 5:**
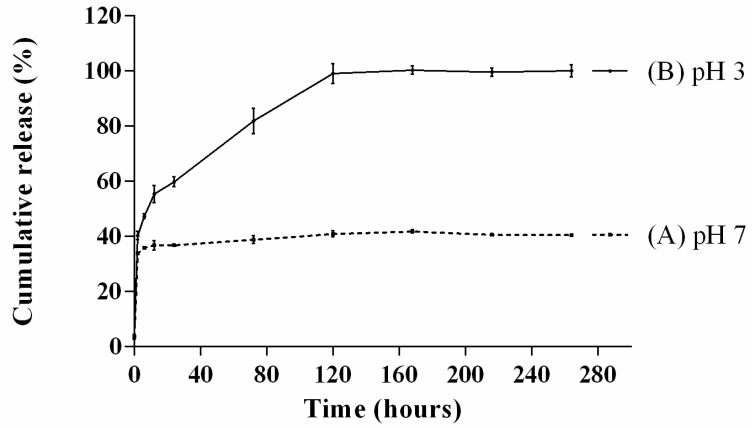
The release profile of BSP at pH 7 (A) and 3 (B) to mimic the condition of physiological environment and endosome/lysosome complex, respectively. At pH 7, BSP was released about 34% from BSP-HAP and no further BSP was released from BSP-HAP thereafter. When in pH 3, BSP-HAP was dissolved and BSP was released completely within the 5 th day (*n* = 6).

The release profile of BSP was evaluated by immersing the synthesized BSP-HAP for a certain period in PBS solutions at pH 7 or pH 3 and is shown in Figure 5. Shown in Figure 5 is the release profile of BSP in PBS solution at pH 7, which mimics physiological pH. An initial burst within 1 h to 34% can be seen; the further release of BSP was not observed. Shown in Figure 5 is the release profile of BSP in PBS solution at pH 3, which mimics the acidic environment of the endosome/lysosome complex; the HAP carrier was dissolved and BSP was released completely within 5 days from the BSP-HAP.

### 3.5. Cytotoxicity Assay and Cell Viability Assay

**Figure 6:**
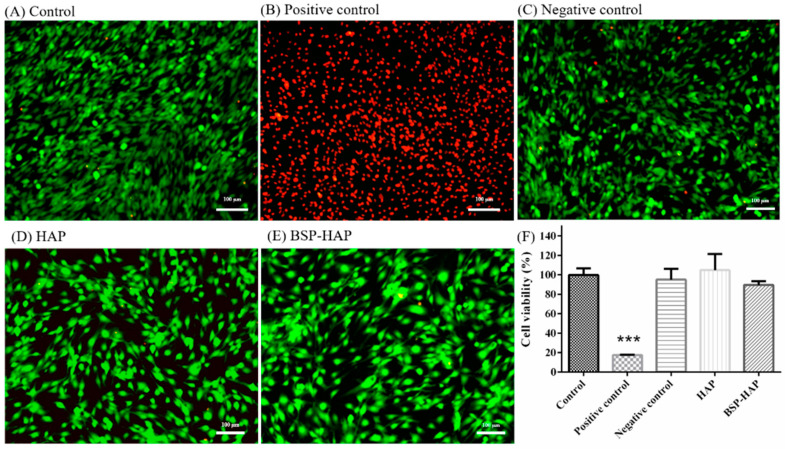
Cytotoxicity assay of BSP-HAP by using LIVE/DEAD staining. (**A**) Control group, (**B**) positive control, (**C**) negative control, (**D**) HAP, and (**E**) BSP-HAP. (**F**) Cell viability evaluation of BSP-HAP by WST-1 assay. C2C12 cultured in DMEM was set as the control group (*n* = 6, *** *p* < 0.001 compared with control). Zinc diethyldithiocarbamate and aluminum oxide served as positive control and negative control, respectively.

The cytotoxic effect of HAP and BSP-HAP was evaluated by using LIVE/DEAD staining. As shown in Figure 6, the dead cells are shown in red and the living cells in green. No significant difference in the ratio of red and green signals among the experimental groups was observed. We infer that the synthesized HAP or BSP-HAP has no cytotoxic potential to C2C12 myoblasts.

As shown in Figure 6F, the effect of HAP and BSP-HAP on cell viability was evaluated by using WST-1 assay. The cell viability of the control group was defined as 100%. The difference in cell viability between the control group and HAP group was less than 9% on average, and the difference in cell viability between the control group and BSP-HAP group was less than 10%. We infer that the synthesized HAP or BSP-HAP would change neither the cell viability nor the mitochondrial activity of C2C12 myoblasts.

### 3.6. Antioxidant Effect of BSP-HAP

**Figure 7:**
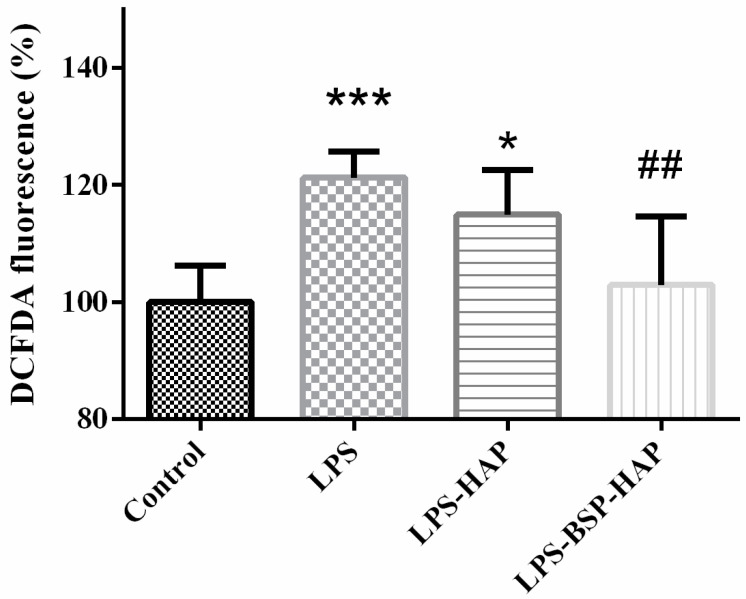
Cellular reactive oxygen species (ROS) generation was measured by DCFDA assay. The results confirmed that BSP-HAP has good antioxidant effect. (*n* = 6, * *p* < 0.05, *** *p* < 0.001 compared with control; ## *p* < 0.01 compared with lipopolysaccharide (LPS)).

The antioxidant effect of BSP-HAP was determined by DCFDA assay, as shown in Figure 7. C2C12 cultured in DMEM was set as the control group and defined as 100%. The ROS level in LPS-HAP group has no antioxidant effect. The ROS level in LPS-BSP-HAP group was 19% lower than the LPS group. Meanwhile, there was no significant difference in ROS levels between the control group and BSP-HAP group. The results confirmed that BSP-HAP has a good antioxidant effect.

### 3.7. Muscle Endurance Analysis by Treadmill Test and Grip Strength Measurement

**Figure 8:**
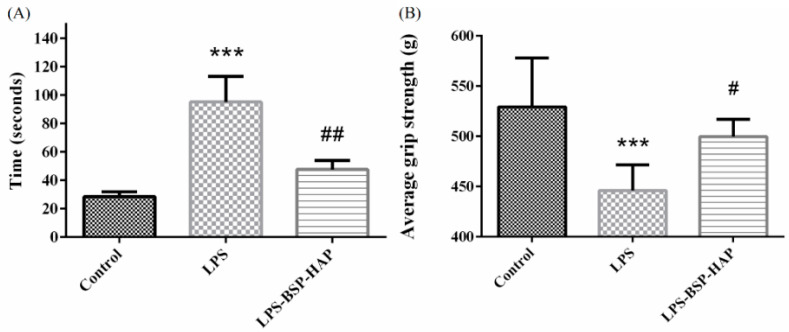
Muscle endurance analysis by using treadmill test (**A**). Average grip strength was measured by using a grip strength meter (**B**). (*n* = 6, *** *p* < 0.001 compared with control group; # *p* < 0.05 compared with LPS group; ## *p* < 0.01 compared with LPS group). From these results, we infer that the muscle endurance and grip strength of rats with LPS-induced sarcopenia and treated with BSP-HAP for 2 months may fully recover.

The treadmill test was performed to evaluate the muscle endurance of rats with LPS-induced sarcopenia. The treadmill test scores of the rats for 2 months are shown in Figure 8A. The number of low performance received by the control, LPS and LPS-BSP-HAP group was approximately 28.5, 95.1, and 47.6 s, respectively, per 30-min treadmill test. These results support that the rats with LPS-induced sarcopenia and IM injected with BSP-HAP for 2 months may effectively increase their muscle endurance.

The results of grip strength measurement are shown in Figure 8B. The average grip strength measured in grams of rats in the control, LPS, and LPS-BSP-HAP groups was 529.3 g, 446.0 g, and 499.7 g, respectively. From these results, we infer that the grip strength of rats with LPS-induced sarcopenia and treated with BSP-HAP for 2 months may fully recover.

### 3.8. The Ratio of Fat Mass to Lean Mass in Rat Muscle by MRI

**Figure 9:**
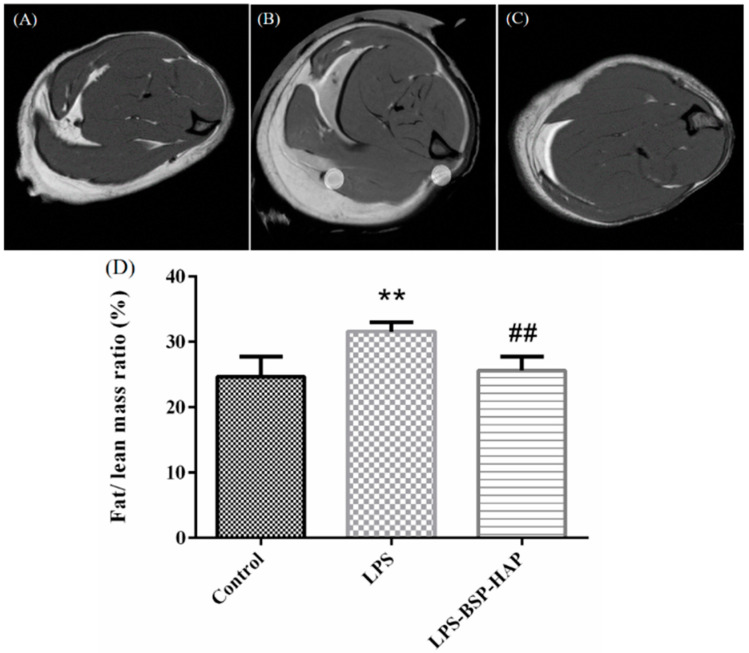
The images from MRI of muscle cross-section (**A**) control group, (**B**) LPS group and (**C**) LPS-BSP-HAP group. The fat and lean mass ratio was calculated by image J (**D**) (*n* = 6, ** *p* < 0.05 compared with control; ## *p* < 0.05 compared with LPS). The ratio of fat mass to lean mass in the muscles of the control, LPS, and LPS-BSP-HAP groups was 24%, 31%, and 25%, respectively.

The cross-sections of rat muscles were examined by MRI and are shown in Figure 9. The white and black areas in the image represent the fat mass and the lean mass in the muscle, respectively; the ratio of fat mass to lean mass was calculated by using Image J software. The ratio of fat mass to lean mass in the muscles of the control, LPS, and LPS-BSP-HAP groups was 24%, 31%, and 25%, respectively. The results indicate that there was no significant difference between the fat/lean mass ratio of the control group and of the LPS-BSP-HAP group.

### 3.9. Safety of BSP-HAP in Vivo by Serological Analysis

The concentration levels of TP, CK, LDH and Ca in serum were analyzed and are shown in Table 3. The values beyond the reference range (as obtained from the control group) are shown in red color and were indicated as abnormal. In the LPS group, the values of CK and LDH were beyond the normal range. The values of TP, CK, LDH and Ca values of the LPS-BSP-HAP group were within the normal range. 

## 4. Discussion

In this study, we extracted and purified 19.8 g BSP from 100 g dried *Bletilla striata* by using a modified extraction method. The extracted BSP was characterized by NMR as shown in Figure 2. We determined that BSP was mainly composed of (1 → 2)-linked α-mannopyranose and (1 → 4)-linked β-D-glucopyranose; this is consistent with a previous study [33].

In this study, we developed a new process at low temperature to synthesize HAP as BSP carrier for sarcopenia that could create more space for BSP loading. The FTIR spectrum of BSP-HAP showed all the absorption bands that corresponded to that of HAP and BSP, as shown in Figure 2C. The crystallinity in BSP-HAP was lower than the traditional HAP due to the low temperature method and BSP loading, as shown in Figure 2. The previous study reported the lower crystallinity HAP has more biocompatibility for long-term use in the body [34]. In a previous study, most of the synthesized HAP exhibited a needle-like shape in the c-axis and along the long-axis with a long-axis-to-short-axis ratio of approximately 6.67 ± 2.83. In the current study, the long-axis was in the range of 2.85 ± 2.46; this is shorter than that in a previous study (Figure 3). We believe that this might be due to a steric separation in BSP macromolecules that limit crystal growth along the long-axis [18]. Because BSP interfere with HAP crystallinity and crystal growth, the BET analysis of BSP-HAP showed higher surface area in comparison to the commercialized HAP (Table 2), resulted in better BSP loading. The size of the synthesized BSP-HAP particles ranges from 500 to 1500 nm; this range is adequate for endocytosis. The efficiency of BSP entrapment in BSP-HAP was calculated by using the following formula: entrapment efficiency (%) = (the amount of BSP in BSP-HAP/total BSP in the system) × 100%. The BSP entrapment efficiency of BSP-HAP was approximately 54% (Figure 4).

The release profile of BSP was determined by immerse BSP-HAP particles in PBS solution with pH value of 3 and 7 to mimic the endosome/lysosome complex and physiological environment, respectively. The results are shown in Figure 5. BSP-HAP immersed in PBS with pH value of 3 would be completely dissolved and BSP would be 100% released within 4 days. We could expect that the BSP-HAP would be dissolved in the acidic environment of endosome/lysosome complex and release BSP completely. On the contrary, when BSP-HAP was immersed in PBS with pH value of 7, BSP release with initial burst occurred in the first few hours due to physical adsorption, followed by a plateau. We believe that BSP will not be released in the physiological environment owing to BSP retained in the HAP carrier. The images from confocal microscope confirmed that the BSP-HAP particles could be engulfed by RAW-264.7 macrophage through the endocytic process (Figure S1). We also infer that BSP-HAP has good biocompatibility as demonstrated in the WST-1 assay and LIVE/DEAD staining (Figure 6).

The age-associated changes to the immune system, immune senescence, oxidative stress and chronic inflammation, have been suggested as major contributors to sarcopenia [3]. However, most of the studies on sarcopenia used young animals induced by different substances, owing to the limited availability and very high costs of using aged animals [35]. Lipopolysaccharide, one of the major molecular components on the outer membrane of Gram-negative bacteria, can cause a dysregulated inflammatory response [36]. In this study, muscle injury and bodyweight reduction are similar to sarcopenia symptoms induced by the LPS-challenged rat model [37]. Meanwhile, the LPS-challenged rat showed lower muscle endurance and strength. LPS-challenged rats mimic sarcopenia muscle, characterized by an increase in intermuscular adipose tissue infiltrations and a decrease in the number and the size of muscle fibers (Figure S2). Increased amounts of intermuscular adipose tissue are correlated to cardiovascular risk. High serum LDH activity is a marker of cell damage [38]. Serum CK is an indicator of muscle degradation; CK levels are highly sensitive to muscle injury, and can therefore be used as a tool to diagnose muscle damage [32,39]. The increase in LDH and CK levels of sarcopenia-like rats could be detected by serological analysis described in Table 3. Blood element analysis results indicated no sign of chronic toxicity in BSP-HAP (Table S1).

In the present study, BSP-HAP delivered by IM administration would stay in the muscle tissue, followed by a gradual uptake via endocytosis. The endosome with BSP-HAP would later be merged with the lysosome as an endosome/lysosome complex, where the HAP could be fully dissolved in minutes due to an acidic environment. Once HAP is dissolved, the endosome/lysosome complex would be broken down because of osmotic pressure from an increase in Ca^2+^ and PO_3_^4−^ in the complex. The high level of Ca^2+^ will circulate in the blood by diffusion. In skeletal muscle fibers, Ca^2+^ plays a crucial role in the excitation-contraction coupling process, which results in action potential of muscle fiber; it is also involved in innumerable functions such as myosin–actin cross bridging, protein synthesis, protein degradation, fiber type shifting, calcium-regulated proteases, and transcription factors. Recent evidence implicates Ca^2+^ dysregulation as a common underlying phenomenon in the pathophysiology of muscles such as sepsis, cachexia, sarcopenia and heart failure [21].

Via endocytosis, BSP might be released into the cytoplasm and eventually into the extracellular space due to a high level of Ca^2+^; BSP might enter the circulatory system by diffusion and be transported throughout the body. In previous studies, *Bletilla striata* polysaccharide has been found have good anti-inflammatory and antioxidant effects to play important roles in endothelial cell proliferation, inducible nitric oxide stimulation, wound healing acceleration and other processes [14,23]. The antioxidant effect was also confirmed that the developed BSP-HAP could suppress LPS-induced ROS generation (Figure 7). We believe that BSP-HAP through BSP exerted anti-oxidation effects and promoted tissue repair in the recovery of LPS-induced muscle injury.

## 5. Conclusions

In this study, we successfully isolated and purified BSP from dry *Bletilla striata* by a modified extraction method to achieve a high yield of almost 20%. The molecular structure and characterized functional groups were identified and confirmed by NMR and FTIR, respectively; those were in agreement with previous reports. The size of the synthesized BSP-HAP particles falls in the range of 500–1500 nm, which was an adequate size for uptake by cells through the endocytosis pathway. The loading capacity of BSP in BSP-HAP was as high as 13.6% by weight ratio. The release profile of BSP from BSP-HAP in vitro proved that the BSP would not be further released in the physiological environment after the initial burst. Instead, the BSP could completely release and escape from the endosome/lysosome complex. BSP-HAP has a good antioxidant effect and the ability to reduce oxidative stress. The results of the animal study showed that the rats treated with BSP-HAP by IM administration could effectively recover from LPS-induced sarcopenia; no matter in muscle endurance by treadmill, grip strength by grip strength meter, or fat/lean mass ratio by MRI. The bio-safety in vivo was proved via blood element analysis and serological analysis after BSP-HAP treatment for a long period. We believe that BSP-HAP delivered by IM administration has great potential for application in sarcopenia therapy and prevention in the future.

## Figures and Tables

**Table 1:** Chemical composition of HAP and BSP-HAP from energy dispersive spectrophotometry (EDS) analysis by atomic ratio. The molar ratio of calcium to phosphorus in the BSP-HAP was 1.55.

Sample	C Atomic %	O Atomic %	P Atomic %	Ca Atomic %
HAP	17.92	53.78	10.62	17.69
BSP-HAP	34.20	49.03	7.24	9.53

**Table 2:** Brunauer, Emmett, and Teller (BET) surface area of BSP-HAP. Where the BET surface area of BSP-HAP was 60.7645 m^2^/g.

Sample	Result
BSP-HAP	BET Surface Area: 60.7645 ± 0.7091 m^2^/g
	Correlation Coefficient: 0.9994673
	Langmuir Surface Area: 96.2750 ± 2.4493 m^2^/g
	Correlation Coefficient: 0.997421

**Table 3:** The blood biochemical analysis serum concentration of total protein (TP), creatine kinase (CK), lactate dehydrogenase (LDH) and calcium (Ca) (*n* = 6, * *p* < 0.05 compared with control; # *p* < 0.05 compared with LPS). Reference: Charles River Laboratories, CD^®^ IGS Rat Model Information Sheet.

	Control	LPS	LPS-BSP-HAP	Reference
Total protein (g/dL)	7.0 ± 0.3	7.4 ± 0.1	6.9 ± 0.2	6.6 ± 1.0
Creatine kinase (U/L)	130.9 ± 23.4	958.3 ± 200.6 *	148.5 ± 13.4 #	131.3 ± 66.4
LDH (U/L)	397.0 ± 77.6	759.4 ± 85.0 *	329.3 ± 95.5 #	500.0 ± 200.0
Calcium (mg/dL)	9.7 ± 0.1	9.8 ± 0.1	9.7 ± 0.2	9.5 ± 0.9

## Data Availability

Data is contained within the article or supplementary material.

## Supplementary Materials

The following are available online at https://www.mdpi.com/2076-3921/10/3/488/s1, Figure S1: The images from confocal microscope showed the distribution of BSP-HAP particles in RAW-264.7 macrophage. Table S1: Safety of BSP-HAP in vivo by blood element analysis. Figure S2: Histological analysis of the muscle fiber size with hematoxylin and eosin staining.
