# Supplementary material for: To Synthesize Hydroxyapatite by Modified Low Temperature Method Loaded with Bletilla striata Polysaccharide as Antioxidant for the Prevention of Sarcopenia by Intramuscular Administration
Source: Antioxidants (Basel). 2021 Mar 20;10(3):488. doi: 10.3390/antiox10030488 (PMC8035982; doi:10.3390/antiox10030488)
Supplement: Supplementary file 1 [file antioxidants-10-00488-s001.zip › Supplementary data-antioxidants/2021-0320-Supplementary data.docx]

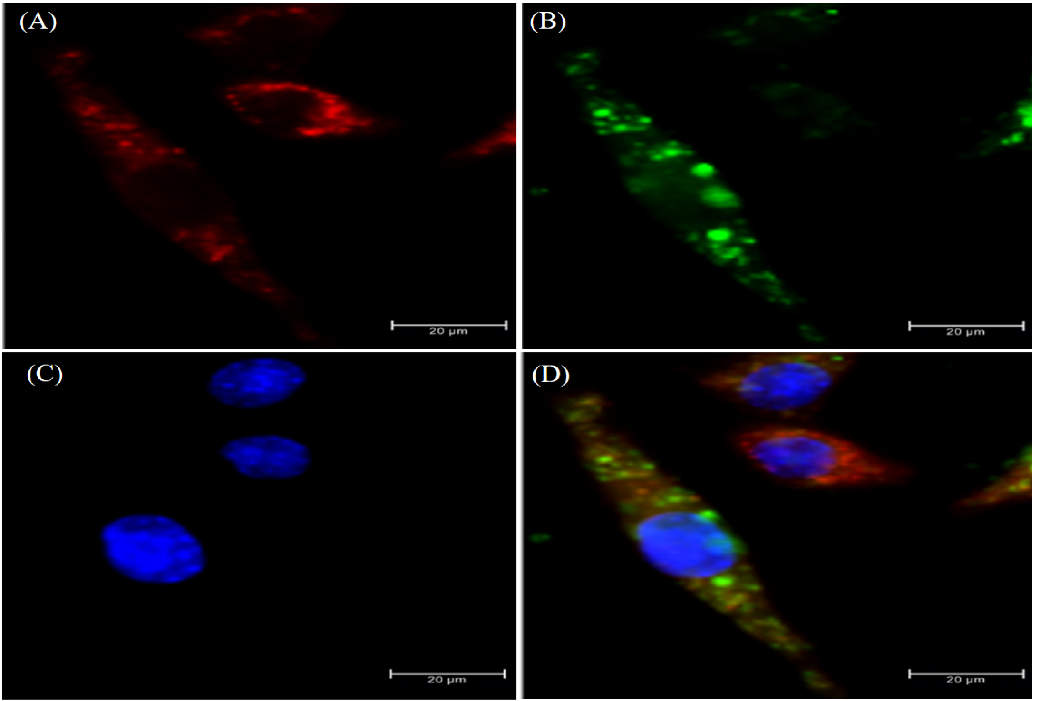


Figure S1: The images from confocal microscope showed the distribution of BSP-HAP particles in RAW-264.7 macrophage. The images from confocal microscope showed the distribution of BSP-HAP particles in RAW-264.7 macrophage. (A) Lysosomes were stained in red by Lyso-Tracker Red. (B) Fluorescein-incorporated BSP-HAP were stained in green. (C) Nuclei were stain in blue by Hoechst 33342. (D) Merged image of all stains. Scale bar was 20 μm. The results confirmed that the BSP-HAP particles could be engulfed by RAW-264.7 macrophage through the endocytic process.

Table S1: Safety of BSP-HAP in vivo by blood element analysis. Reference: Charles River Laboratories, CD® IGS Rat Model Information Sheet. All results indicated no sign of chronic toxicity in BSP-HAP.

|  | Control | LPS | LPS-BSP-HAP |
| --- | --- | --- | --- |
| RBC (M/µL) | 8.56 ± 0.63 | 8.42 ± 0.22 | 8.33 ± 0.48 |
| HGB (g/dL) | 15.33 ± 1.10 | 14.50 ± 0.52 | 14.76 ± 0.65 |
| HCT (%) | 45.93 ± 4.63 | 43.40 ± 2.42 | 43.66 ± 2.50 |
| MCV (fL) | 53.50 ± 1.57 | 51.53 ± 1.53 | 52.43 ± 1.15 |
| MCH (pg) | 17.90 ± 0.10 | 17.23 ± 0.20 | 17.73 ± 0.35 |
| MCHC (g/dL) | 33.46 ± 1.11 | 33.43 ± 0.70 | 33.80 ± 0.45 |
| RET (K/ µL) | 240.00 ± 72.20 | 236.26 ± 9.75 | 265.93 ± 86.06 |
| PLT (K/ µL) | 1139.66 ± 115.17 | 954.66 ± 79.25 | 1192.66 ± 98.21 |
| WBC (K/ µL) | 9.99 ± 1.97 | 12.30 ± 1.68 | 12.53 ± 2.16 |
| NEUT (%) | 19.16 ± 13.46 | 14.30 ± 1.38 | 14.80 ± 6.15 |
| LYMPH (%) | 72.33 ± 12.67 | 78.93 ± 1.61 | 76.60 ± 4.96 |
| MONO (%) | 4.63 ± 0.49 | 4.03 ± 0.57 | 4.86 ± 0.45 |
| EO (%) | 3.76 ± 1.62 | 2.06 ± 0.72 | 3.60 ± 1.41 |
| BASO (%) | 0.20 ± 0.10 | 0.20 ± 0.10 | 0.13 ± 0.05 |
| RBC: red blood cell; HGB: hemoglobin; HCT: hematocrit; MCV: mean corpuscular volume: MCH: mean corpuscular hemoglobin; MCHC: mean corpuscular hemoglobin concentration; PLT: platelet; WBC: white blood cell; NEUT: neutrophil; LYMPH: lymphocyte; MONO: monocyte; EO: eosinophil; BASO: basophil. | | | |


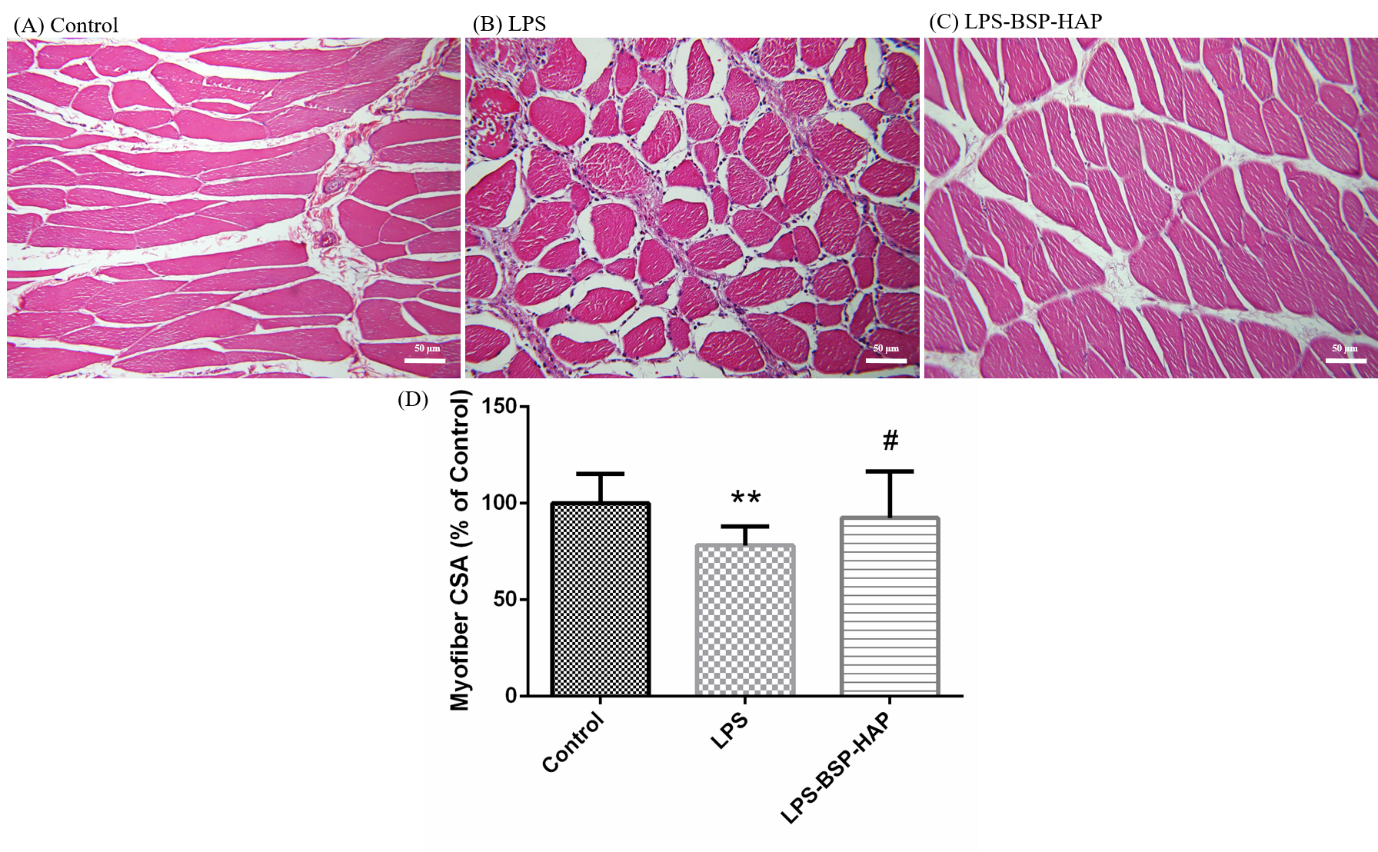


Figure S2: Histological analysis of the muscle fiber size with hematoxylin and eosin staining. Rat were divided into (A) Control group, (B) LPS group, and (C) LPS-BSP-HAP group. (D) Quantification of muscle fibers cross-sectional area (CSA) was calculated by ImageJ software, where control group CSA was set at 100 %. Scale bar = 50 μm. (n = 6, ** P < 0.01 compared with control; # P < 0.05 compared with LPS).
