# Supplementary figures and images for: To Synthesize Hydroxyapatite by Modified Low Temperature Method Loaded with Bletilla striata Polysaccharide as Antioxidant for the Prevention of Sarcopenia by Intramuscular Administration
Source: Antioxidants (Basel). 2021 Mar 20;10(3):488. doi: 10.3390/antiox10030488 (PMC8035982; doi:10.3390/antiox10030488)

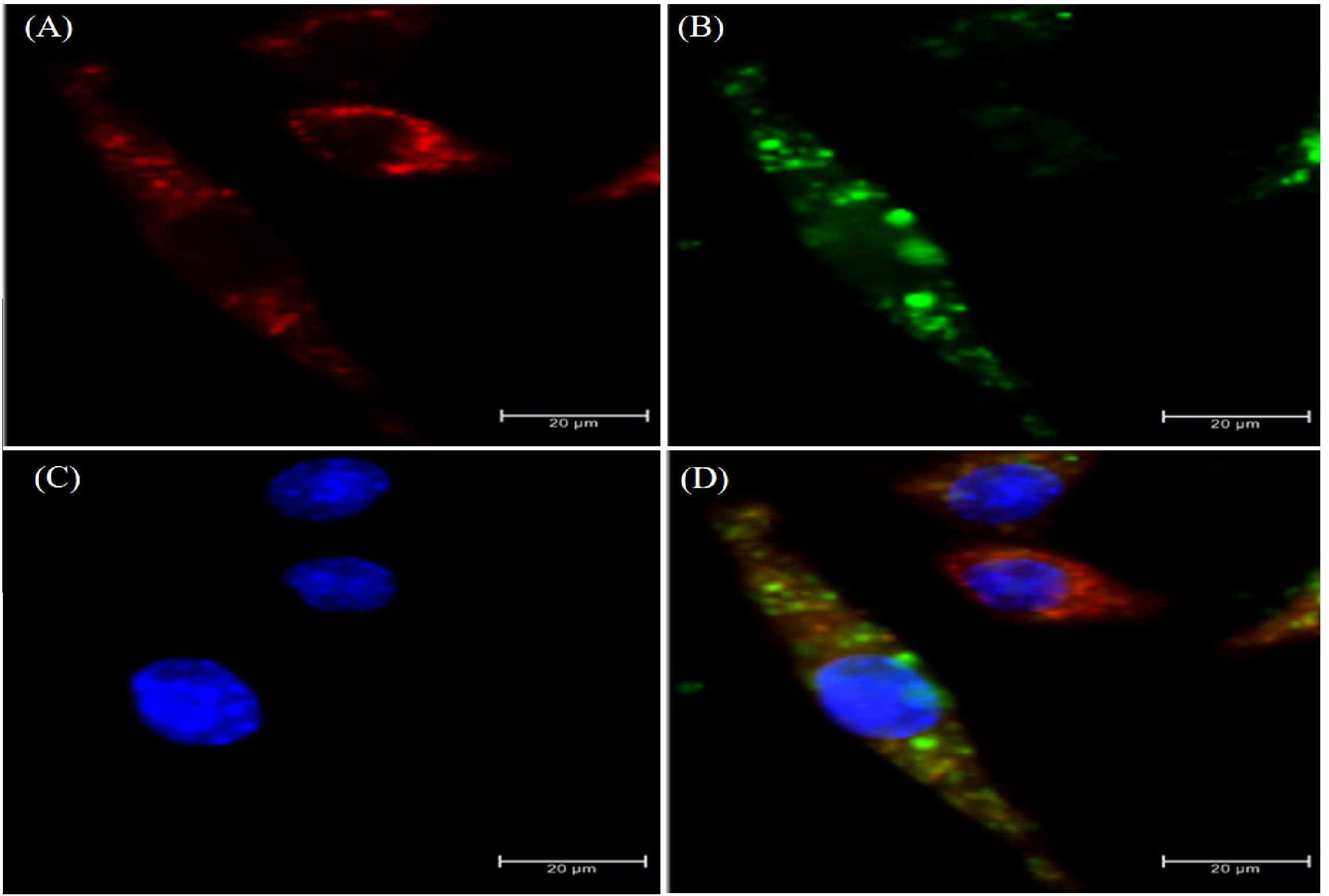

Supplement: Supplementary file 1 [file antioxidants-10-00488-s001.zip › Supplementary data-antioxidants/Figure S1.tif]

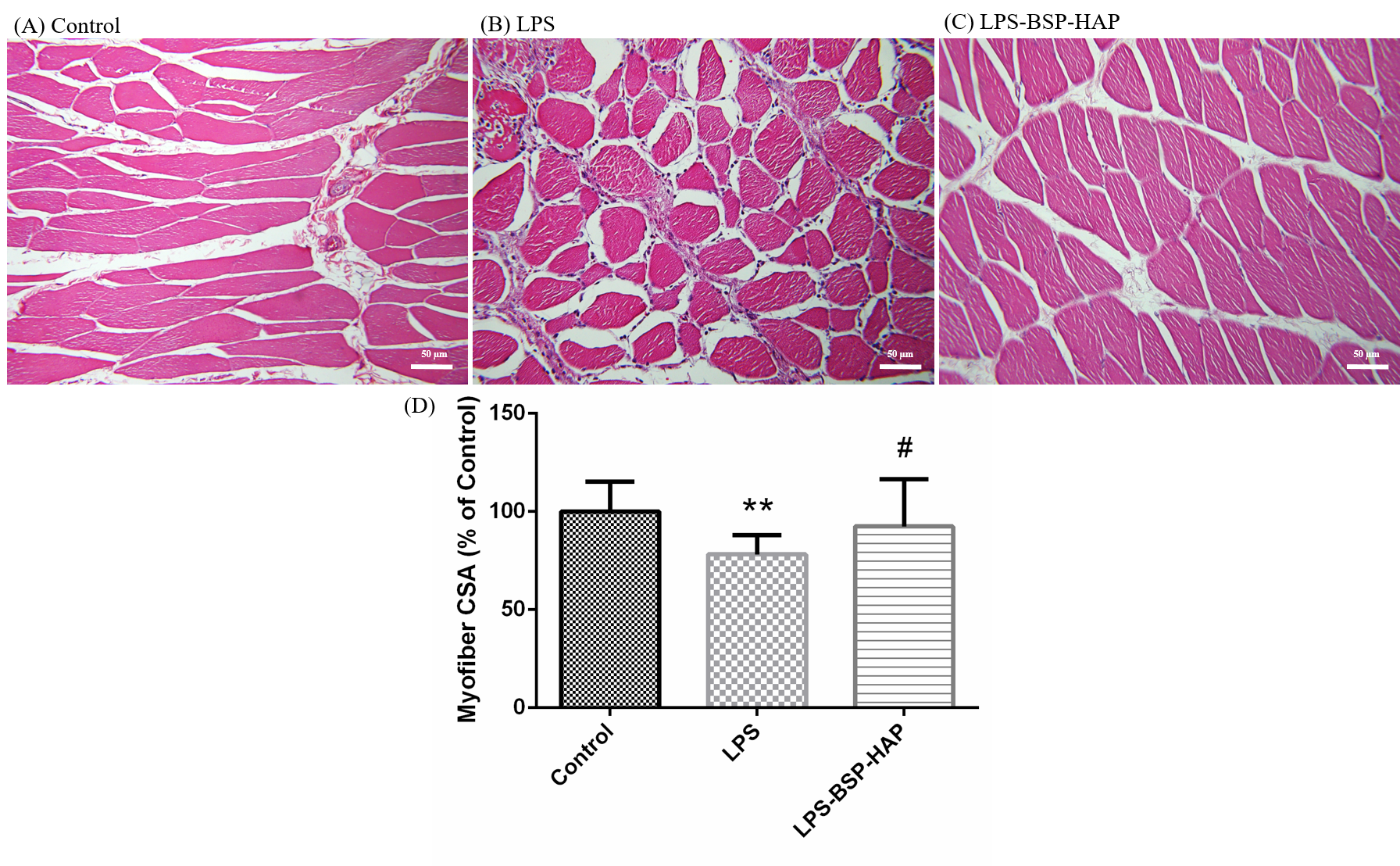

Supplement: Supplementary file 1 [file antioxidants-10-00488-s001.zip › Supplementary data-antioxidants/Figure S2.tif]

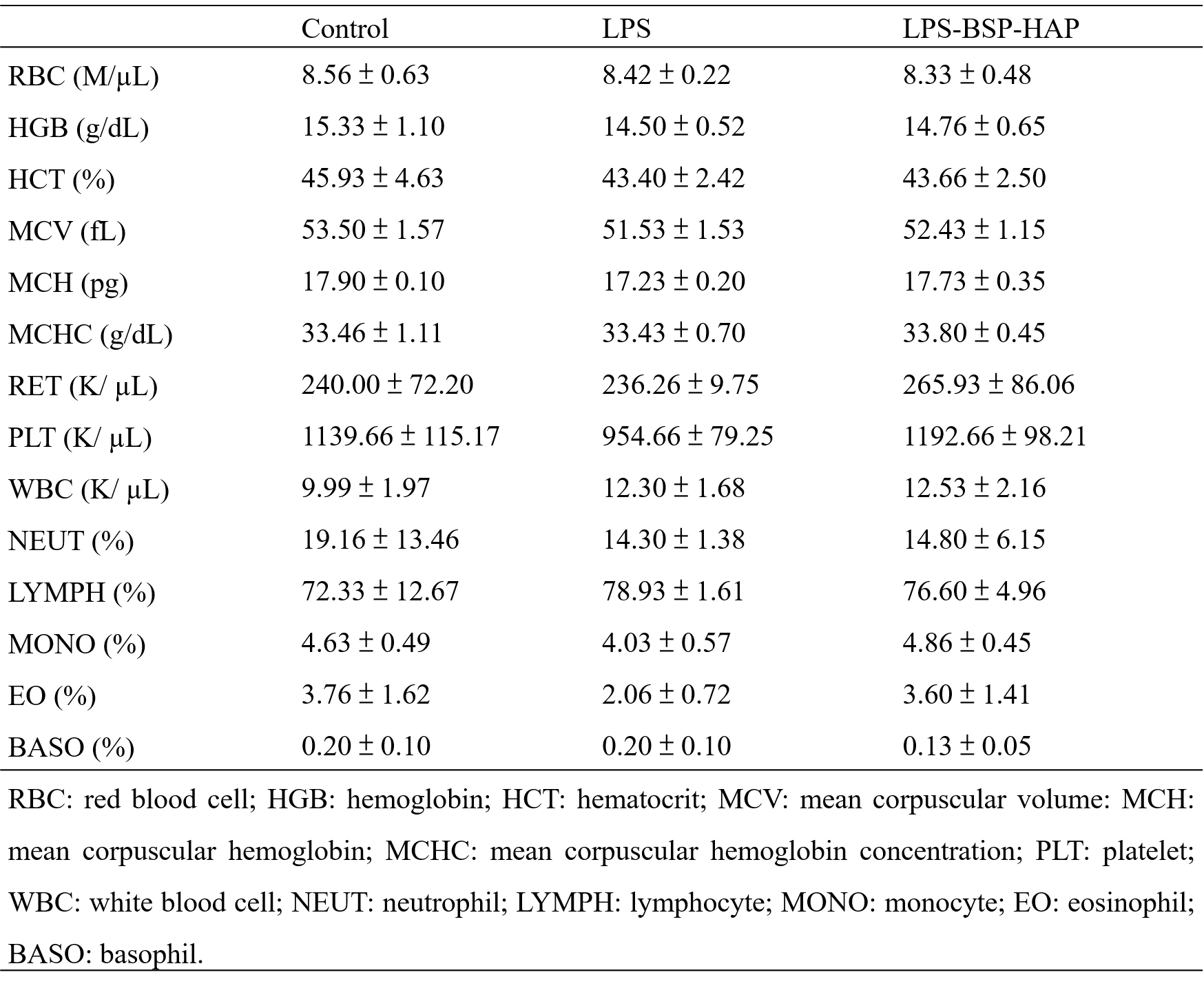

Supplement: Supplementary file 1 [file antioxidants-10-00488-s001.zip › Supplementary data-antioxidants/Table S1.tif]
